# Endometriosis and adverse pregnancy outcomes in primiparous women: a retrospective cohort study

**DOI:** 10.1007/s00404-026-08465-5

**Published:** 2026-05-20

**Authors:** Julia Téoule, Leonie Zikarsky, Karin Bundschu, Sophia Andres, Katharina Hancke

**Affiliations:** Department of Gynecology and Obstetrics, Ulm University Hospital, Prittwitzstraße 43, 89075 Ulm, Germany

**Keywords:** Medically assisted reproduction (MAR), Endometriosis, Placenta previa, Pregnancy outcomes, Primiparity

## Abstract

**Objective:**

To assess whether pre-existing endometriosis is associated with specific adverse pregnancy and delivery outcomes in primiparous women.

**Design:**

Retrospective cohort study.

Setting.

Tertiary care university hospital.

Population or sample.

Primiparous women with singleton pregnancies.

**Methods:**

Women with endometriosis were compared with primiparous controls without endometriosis. Primary outcomes were preterm birth and cesarean delivery; placenta previa was examined as a key secondary outcome. Multivariable logistic regression models adjusted for maternal age and mode of conception were used.

Main outcome measures.

Preterm birth, cesarean delivery, placenta previa.

**Results:**

Among 16,033 primiparous women, 118 had a diagnosis of endometriosis prior to pregnancy. After adjustment for maternal age and mode of conception, endometriosis was not independently associated with preterm birth (adjusted odds ratio 0.75; 95% confidence interval 0.44–1.28) or cesarean delivery (adjusted odds ratio, 1.17; 95% confidence interval, 0.81–1.71). In contrast, endometriosis was associated with placenta previa (adjusted odds ratio, 5.90; 95% confidence interval, 2.37–14.71).

**Conclusion:**

In primiparous women, pre-existing endometriosis was independently associated with placenta previa, but not with preterm birth or cesarean delivery. These findings support abnormal early placentation as a potential mechanism linking endometriosis to adverse obstetric outcomes.

**Supplementary Information:**

The online version contains supplementary material available at 10.1007/s00404-026-08465-5.

## What does this study adds to the clinical work

**Table:** 

Whether endometriosis independently predisposes to placenta previa has been unclear. In this cohort of primiparous women, endometriosis was associated with a substantially increased risk independent of conception mode, though the relatively small number of events limits precision. These findings suggest that careful placental assessment may be warranted in women with endometriosis.	

## Introduction

Endometriosis is a chronic gynecological disease estimated to affect up to 10% of women of reproductive age and is commonly characterized by chronic pelvic pain and infertility [1]. Many affected women nevertheless conceive, and emerging evidence suggests that endometriosis is associated with an elevated risk of adverse obstetric outcomes, including preterm birth, placenta previa, and cesarean delivery [2–6]. However, interpretation of these associations is complicated by substantial heterogeneity across studies.

In particular, much of the existing evidence is derived from large registry-based studies and meta-analyses that encompass heterogeneous populations, including multiparous women and rely on administrative endometriosis diagnoses ascertained during or after pregnancy, resulting in substantial variability in disease definition and timing across studies [5, 7, 8]. As a result, causal inference remains limited, and it is often unclear whether reported obstetric risks reflect the effect of endometriosis itself or downstream consequences of prior obstetric history.

Furthermore, medically assisted reproduction (MAR) is disproportionately used among women with endometriosis and is independently associated with adverse pregnancy and delivery outcomes, including preterm birth, placenta previa, and cesarean delivery [9–11]. However, conception mode is inconsistently reported or accounted for in many observational studies [5, 7]. Importantly, few studies have specifically examined whether obstetric risks associated with endometriosis are already present in the first pregnancy [12, 13].

The aim of this study was to investigate the association between pre-existing endometriosis and adverse pregnancy and delivery outcomes, with a primary focus on preterm birth and cesarean delivery. In addition, we examined the risk of placenta previa as a key secondary outcome. To isolate the effect of endometriosis itself, analyses were restricted to primiparous women with histologically confirmed endometriosis diagnosed prior to conception.

## Materials and methods

### Study design and setting

This retrospective cohort study was conducted at the Department of Obstetrics and Gynecology, Ulm University Hospital, a certified endometriosis center and level I perinatal center. Women with endometriosis were identified through the hospital information system, based on a documented surgical diagnosis and a subsequent delivery at our institution. All women in the endometriosis group had undergone laparoscopic surgery with histological confirmation of endometriosis prior to their first delivery. Only primiparous women were included, defined as women with no prior live births or stillbirths before the index pregnancy. Multiple gestations were excluded. Deliveries in the endometriosis group occurred between April 2004 and June 2025, with prior surgical treatment for endometriosis performed between March 2000 and March 2024. The control group consisted of primiparous women without a diagnosis of endometriosis who delivered singleton pregnancies at the same institution between January 2014 and June 2025. Controls were identified from the institutional obstetric database and had no documented clinical or surgical diagnosis of endometriosis. The different inclusion periods reflect a transition in the hospital documentation system, which limited availability of complete obstetric data for controls prior to 2014.

### Exposure and covariates

The exposure of interest was endometriosis, defined as laparoscopically and histologically confirmed disease diagnosed prior to the index pregnancy. In a small minority of women (*n* = 5), endometriosis was diagnosed intraoperatively at the time of cesarean delivery and was therefore presumed to have been present prior to pregnancy. Endometriosis phenotype was classified based on surgical reports as peritoneal endometriosis, ovarian endometrioma, and/or deep infiltrating endometriosis. Where available, endometriosis severity was classified according to the revised American Society for Reproductive Medicine (rASRM) classification and the #Enzian classification based on the original surgical reports [14, 15]. Maternal age at delivery was included as a continuous variable in adjusted analyses. Body mass index (BMI) was incompletely documented and therefore not included in the primary multivariable models. Mode of conception was categorized as spontaneous conception or MAR.

### Outcome measures

The primary outcomes were preterm birth and cesarean delivery. Preterm birth was defined as delivery before 37 + 0 weeks of gestation. Placenta previa was examined as a secondary outcome and was defined as placenta previa confirmed intraoperatively at the time of delivery. Gestational age at delivery was determined based on first-trimester ultrasound or last menstrual period.

### Statistical analysis

Baseline characteristics were summarized using descriptive statistics. Categorical variables were compared using Chi-square tests, and continuous variables using the Mann–Whitney U test. Continuous variables are presented as medians with interquartile ranges (IQR). Associations between endometriosis and obstetric outcomes were assessed using binary logistic regression models, with results presented as odds ratios and 95% confidence intervals. Primary multivariable models were adjusted for maternal age at delivery and MAR. Model calibration was evaluated using the Hosmer–Lemeshow goodness-of-fit test. Data completeness was assessed for all covariates prior to analysis. Body mass index (BMI) was incompletely documented and therefore not included in the primary multivariable models. Complete-case analyses were performed for all primary analyses.

To determine whether the association between endometriosis and placenta previa differed by mode of conception, a two-way interaction term between endometriosis and MAR was included in the multivariable logistic regression model.

As adenomyosis was present in a subset of the endometriosis cohort (*n* = 29; 24.6%) and may independently contribute to abnormal placentation, a prespecified sensitivity analysis was performed excluding women with documented adenomyosis to assess whether the observed association between endometriosis and placenta previa persisted in the absence of this comorbidity. No imputation methods were applied due to the retrospective nature of the study. All statistical analyses were performed using IBM SPSS Statistics for Mac (version 31.0.0.0; IBM Corp., Armonk, NY). A two-sided *P* value < 0.05 was considered statistically significant.

Given the retrospective design, no core outcome set was applicable, and patients or the public were not involved in the design, conduct, or reporting of the research.

### Ethics approval

This retrospective cohort study was approved by the Ethics Committee of Ulm University (approval number 197/25). Due to the retrospective nature of the study and the use of anonymized clinical data, the requirement for informed consent was waived.

### Strobe statement

This study is reported in accordance with the Strengthening the Reporting of Observational Studies in Epidemiology (STROBE) guidelines.

## Results

### Study population

A total of 16,033 primiparous women with singleton pregnancies were included in the analysis, comprising 118 women with histologically confirmed endometriosis diagnosed prior to conception and 15,915 controls without endometriosis. All deliveries occurred at Ulm University Hospital. Women in the endometriosis group had undergone laparoscopic surgery for endometriosis before their first delivery. Multiple gestations were excluded.

### Baseline characteristics

Baseline characteristics are shown in Table 1. Women with endometriosis were significantly older at delivery compared with controls (median age, 32 vs 31 years; *P* < 0.001). Conception by MAR was substantially more common among women with endometriosis (41.5% vs 4.4%; *P* < 0.001). Documented pre-pregnancy BMI index did not differ significantly between groups (median (IQR), 24.0 (21.5–27.1) vs 23.4 (21.1–27.0); *P* = 0.443). Clinical characteristics of the endometriosis cohort are shown in Table S1.

**Table 1:** Baseline characteristics of primiparous women with and without endometriosis

Characteristic	Endometriosis (*n* = 118)	Controls (*n* = 15,915)	*P* value
Maternal age at delivery, years, median (IQR)	32 (30–36)	31 (27–34)	< 0.001
Pre-pregnancy BMI, kg/m^2^, median (IQR)*	24.0 (21.5–27.1)	23.4 (21.1–27.0)	0.443
Medically assisted reproduction, *n* (%)	49 (41.5)	694 (4.4)	< 0.001

Data are presented as median (IQR) or n (%). *P *values were calculated using the Mann–Whitney *U* test for continuous variables and Chi-square test for categorical variables. *Body mass index was available for 97 women with endometriosis and 14,892 controls. *BMI* body mass index, *IQR* interquartile range

### Pregnancy and delivery outcomes

Unadjusted pregnancy and delivery outcomes are summarized in Table 2. Cesarean delivery rates were high in both groups (41.5% and 32.7%, Table 2), consistent with the tertiary referral setting. Cesarean delivery occurred more frequently among women with endometriosis than among controls (41.5% vs 32.7%; *P* = 0.041). In the endometriosis group, this corresponded to a total of 49 cesarean deliveries. Indications included placenta previa (*n* = 6), non-cephalic presentation (*n* = 5), and primary cesarean delivery for maternal indications (*n* = 3). The majority of procedures were secondary cesarean deliveries-decided intrapartum (*n* = 31). Cesarean delivery on maternal request accounted for three cases. Rates of preterm birth before 37 weeks of gestation were similar between groups (13.6% vs 14.3%; *P* = 0.813). The distribution of gestational age subgroups among preterm births is shown in **Table S4**. Placenta previa was significantly more common in the endometriosis group (5.1% vs 0.5%; *P* < 0.001). Additional pregnancy and neonatal outcomes, including birthweight, neonatal sex, and gestational diabetes, are presented in **Table S2**.

**Table 2:** Pregnancy and delivery outcomes in primiparous women with and without endometriosis

Outcome	Endometriosis (*n* = 118)	Controls (*n* = 15,915)	*P* value
Gestational age at delivery, days, median (IQR)	276 (267–283)	276 (268–283)	0.504
Preterm birth < 37 weeks, *n* (%)	16 (13.6)	2280 (14.3)	0.813
Cesarean delivery, *n* (%)	49 (41.5)	5198 (32.7)	0.041
Placenta previa, *n* (%)	6 (5.1)	83 (0.5)	< 0.001
Placental abruption, *n* (%)	2 (1.7)	68 (0.4)	0.037
Fetal growth restriction, *n* (%)	13 (11.0)	823 (5.2)	0.004
Gestational hypertension, *n* (%)	5 (4.2)	129 (0.8)	< 0.001
Pre-eclampsia, *n* (%)	9 (7.6)	815 (5.1)	0.219

Data are presented as median (IQR) or n (%). *P* values were calculated using the Mann–Whitney *U* test for continuous variables and Chi-square test or Fisher’s exact test, as appropriate. *IQR* interquartile range

### Multivariable analyses

Results of adjusted logistic regression analyses are presented in Table 3. After adjustment for maternal age at delivery and MAR, endometriosis was not independently associated with cesarean delivery (aOR 1.17; 95% CI 0.81–1.71) or preterm birth (aOR 0.75; 95% CI 0.44–1.28). In contrast, endometriosis was strongly associated with placenta previa, independent of maternal age and MAR (aOR 5.90; 95% CI 2.37–14.71).

**Table 3:** Adjusted association between pre-existing endometriosis and obstetric outcomes

Outcome	Adjusted OR (95% CI)	*P* value
Cesarean delivery	1.17 (0.81–1.71)	0.404
Preterm birth < 37 weeks	0.75 (0.44–1.28)	0.286
Placenta previa	5.90 (2.37–14.71)	< 0.001

Binary logistic regression models adjusted for maternal age at delivery and medically assisted reproduction. Endometriosis was defined as laparoscopically and histologically confirmed disease diagnosed prior to conception. Model calibration for the placenta previa model was adequate (Hosmer–Lemeshow *χ*^2^ = 11.261; df = 8; *P* = 0.187). *CI* confidence interval, *OR* odds ratio

### Effect modification by mode of conception

To determine whether mode of conception modified the association between endometriosis and placenta previa, we fitted an additional multivariable logistic regression model including a two-way interaction term between endometriosis and MAR (endometriosis × MAR), with adjustment for maternal age (Table 4). The interaction term was not statistically significant (aOR for interaction 0.97; 95% CI 0.15–6.23; *P* = 0.976), providing evidence that the direction and strength of the endometriosis–placenta previa association did not differ between spontaneously conceived pregnancies and those conceived by MAR. In this model, endometriosis remained independently associated with placenta previa (aOR 6.00; 95% CI 1.44–24.99; *P* = 0.014).

**Table 4:** Interaction between endometriosis and mode of conception in relation to placenta previa

Predictor	Adjusted OR	95% CI	*P* value
Endometriosis	6.00	1.44–24.99	0.014
MAR	2.59	1.33–5.04	0.005
Endometriosis × MAR	0.97	0.15–6.23	0.976
Maternal age at delivery	1.06	1.02–1.11	0.005

Binary logistic regression model including a two-way interaction term between endometriosis and medically assisted reproduction, adjusted for maternal age at delivery. *CI* confidence interval, *MAR* medically assisted reproduction, *OR* odds ratio

### Sensitivity analysis excluding women with adenomyosis

To address the potential confounding effect of adenomyosis, we repeated the primary multivariable analysis after excluding the 29 women (24.6%) with documented adenomyosis. Among the remaining 89 women with endometriosis, 3 had placenta previa (3.4%). The association between endometriosis and placenta previa remained statistically significant (aOR 4.08; 95% CI 1.21–13.75; *P* = 0.023), indicating that the observed association was not solely attributable to coexisting adenomyosis (**Table S3**).

### Sensitivity analysis excluding women with previous pregnancy

In a further sensitivity analysis restricted to women with no previous pregnancy of any kind (endometriosis *n* = 87, controls *n *= 12,572), endometriosis remained associated with placenta previa (aOR 4.29; 95% CI 1.24–14.85; *P* = 0.021), indicating that the observed association is not explained by prior early pregnancy loss.

## Discussion

In this cohort of primiparous women with singleton pregnancies, pre-existing endometriosis was associated with a higher rate of placenta previa, whereas no independent associations were observed with preterm birth or cesarean delivery after adjustment for maternal age and mode of conception.

Key strengths of this study include restriction to primiparous women, a well-defined exposure based on laparoscopic and histological confirmation of endometriosis prior to conception, and detailed clinical data from a single tertiary center. Together, these features minimize confounding by prior obstetric history and enhance internal validity. Limitations include the small number of placenta previa events (*n* = 6), resulting in wide confidence intervals and limited precision of effect estimates. The modest sample size precludes subgroup analyses by endometriosis phenotype and limits statistical power for secondary outcomes. Adenomyosis was present in approximately one-quarter of the endometriosis cohort and may independently contribute to abnormal placentation. However, a sensitivity analysis excluding women with adenomyosis yielded similar results, and the association between endometriosis and placenta previa remained directionally consistent after exclusion of this comorbidity. Mode of conception was categorized as spontaneous or MAR, without further distinction between IVF, ICSI, or ovulation induction, which may obscure heterogeneity within the MAR group. Finally, undiagnosed endometriosis among controls cannot be excluded, although any such misclassification would likely bias results toward the null. The long study period (surgical treatment 2000–2024, deliveries 2004–2025) reflects the need to assemble a sufficiently large clinically phenotyped cohort of primiparous women with histologically confirmed endometriosis, and is associated with changes in endometriosis management over time. However, the exposure definition used here—laparoscopic and histological confirmation of endometriosis prior to the index pregnancy—remained stable throughout the period and is widely regarded as the diagnostic reference standard [16]. Furthermore, all six placenta previa events occurred in women who delivered between 2014 and 2025, i.e., within the more recent treatment era. Formal staging according to rASRM and #Enzian was available only for a subset of women, reflecting the fact that the #Enzian classification was only introduced in 2005 and formally updated in 2021 [15]. Although the small number of cases precluded a formal statistical analysis of the association between disease severity and placenta previa, the descriptive distribution showed a predominance of deep infiltrating and advanced disease features among women with placenta previa. This finding is consistent with previous reports of increased placenta previa in women with advanced endometriosis [17].

From a clinical perspective, these findings indicate that endometriosis represents an independent risk for placenta previa, irrespective of conception mode. Current international guidelines focus on established maternal risk factors for placenta previa, such as prior cesarean delivery, advanced maternal age, and MAR, but do not explicitly include endometriosis among guideline-defined risk determinants [18, 19]. Nevertheless, the present findings support the hypothesis that endometriosis may constitute an additional uterine risk phenotype predisposing to low placental implantation. As placental location is routinely assessed at the mid-pregnancy anomaly scan, heightened attention to placental localization may be warranted for women with endometriosis [19]. If placenta previa is identified, management typically includes follow-up imaging with transvaginal ultrasound to reassess placental position and to guide individualized counseling, surveillance, and delivery planning [19]. Beyond placenta-related considerations, the absence of independent associations with other obstetric outcomes suggests that routine obstetric management may not require modification solely on the basis of an endometriosis diagnosis.

These observations are biologically plausible and consistent with well-described alterations in endometrial receptivity, decidualization, and uterine function associated with endometriosis [9, 20–23]. As all women were primiparous, the strongest established risk factor for placenta previa—prior cesarean delivery—was absent, indicating that the observed association cannot be attributed to cesarean-related uterine scarring. Endometriosis is characterized by chronic inflammation, progesterone resistance, impaired decidualization, and abnormalities of the myometrial junctional zone, features that may disrupt normal implantation dynamics and trophoblast invasion [9, 20–23]. Perturbed uterine peristalsis and altered implantation site selection have also been proposed as mechanisms linking endometriosis to abnormal placentation [5, 24]. The present findings support the concept of a pre-existing uterine environment predisposing to abnormal placentation.

Consistent with this interpretation, population-based cohort studies and meta-analyses have repeatedly reported an increased risk of placenta previa among women with endometriosis, although effect sizes vary [7, 25–27]. Larger registry-based studies generally demonstrate moderate associations, whereas analyses accounting for conception mode or restricting to spontaneous conceptions tend to report stronger effects [27, 28]. The magnitude observed in the present cohort aligns with estimates from studies minimizing exposure misclassification [5]. The systematic review and meta-analysis by Matsuzaki et al. demonstrated higher pooled effect estimates in adjusted analyses and in subgroups with histologically confirmed endometriosis, supporting the interpretation that reliance on registry-based diagnoses may attenuate observed associations due to dilution of the exposed population [5]. A more recent meta-analysis by Busnelli et al. further extended this evidence by demonstrating a clear biological gradient: effect estimates for placenta previa rose markedly in rASRM stage III–IV endometriosis (OR 6.61) and deep infiltrating endometriosis (OR 14.54) [17].

Several clinically detailed cohort studies with surgically and histologically confirmed endometriosis further contextualize these findings. Lin et al. reported a more than fourfold increased risk of placenta previa (adjusted OR 4.51; 95% CI 1.23–16.50) among nulliparous women with spontaneous singleton pregnancies and preconception histological confirmation of endometriosis [13]. Compared with this cohort, the present study extends existing evidence by explicitly accounting for mode of conception. Vercellini et al. exclusively studied women achieving a first spontaneous pregnancy after surgery for endometriosis and reported that the risk of placenta previa varies by endometriosis phenotype and is highest in women with rectovaginal endometriosis (OR 5.81; 95% CI 1.53–22.03) [12]. However, the absence of a contemporaneous control group without endometriosis limited causal inference regarding endometriosis per se.

Mannini and Sorbi et al. likewise observed higher rates of placenta previa in women with histologically confirmed endometriosis, but parity was mixed and nulliparity was treated as a covariate rather than a defining design feature [29]. More recently, Gruber et al. reported a markedly increased rate of placenta previa in a German case–control study of primiparous women with deep infiltrating endometriosis and coexistent adenomyosis compared with controls matched for maternal age and mode of conception (9.8% vs 1.2%; OR 8.76, 95% CI 1.55–49.61) [30]. A recent narrative review by Brunes et al. examined whether surgery prior to pregnancy in cases of advanced endometriosis affects obstetric risks, and concluded that surgical treatment does not reduce the increased risk of placenta previa in cases of high-grade endometriosis/adenomyosis [31]. This supports the interpretation that the underlying uterine environment in advanced disease, rather than surgical status alone, is a key determinant of placenta-related outcomes. While recent reports have raised the possibility that part of the obstetric risks attributed to endometriosis may in fact be driven by coexisting adenomyosis [17]. However, our sensitivity analysis excluding women with documented adenomyosis showed a persistent association between endometriosis and placenta previa, indicating that endometriosis confers an independent risk irrespective of adenomyosis status.

Taken together, these studies support a biologically consistent association between endometriosis and abnormal placentation, while highlighting the importance of cohort definition, parity restriction, and conception-mode stratification. By restricting the population to primiparous women with a preconception, histologically confirmed diagnosis of endometriosis and explicitly accounting for MAR, the present study reduces key sources of confounding that have limited interpretability of earlier findings.

From a clinical perspective, although infrequent at a population level, the absolute rate of placenta previa among women with endometriosis in the present cohort (5.1%) indicates that this placental phenotype is clinically meaningful and directly relevant to antenatal surveillance and delivery planning. Absolute rates among controls were consistent with population-based estimates, supporting the validity of the reference group [25, 26]. Taken together, these findings suggest that previously reported associations between endometriosis and broader obstetric outcomes may be largely mediated through abnormal placentation, rather than reflecting a generalized obstetric risk attributable to endometriosis itself [27, 28].

In this context, the absence of an independent association between endometriosis and preterm birth in the present analysis contrasts with several large population-based studies reporting modestly increased risks [8, 28]. Ibiebele et al. reported a modest increase in preterm birth among women with endometriosis who conceived without ART compared with controls (adjusted risk ratio (aRR) 1.24; 99% CI 1.06–1.44) [28]. Similarly, Velez et al. observed comparable estimates independent of mode of conception (aRR 1.26; 95% CI 1.20–1.33) [8]. Notably, these studies included mixed-parity populations. Findings from clinical cohorts with histologically confirmed endometriosis are heterogeneous with respect to preterm birth. Lin et al. observed increased rates of preterm birth among nulliparous women with spontaneously conceived singleton pregnancies (adjusted OR 2.42; 95% CI 1.05–5.57) [13].

Mannini and Sorbi et al. reported higher overall rates of preterm birth among women with surgically and histologically confirmed endometriosis (OR 0.32 for the non-event; 95% CI 0.20–0.52); however, estimates were not adjusted for mode of conception, and parity was mixed [29]. In our cohort, overall rates of preterm birth were high in both groups (Table 2), which may have limited the ability to detect an additional effect attributable to endometriosis. Evidence from registry-based cohorts and meta-analyses suggests that preterm delivery in women with endometriosis is frequently associated with placental pathology, particularly placenta previa, rather than reflecting a primary tendency toward spontaneous preterm labor [5, 28]. This interpretation is supported by lesion-specific analyses indicating that elevated preterm birth rates often co-occur with placental abnormalities or disease severity rather than representing a uniform effect of endometriosis across all pregnancies [29, 32].

Similarly, although cesarean delivery was more frequent among women with endometriosis in unadjusted analyses, this association was no longer independent after adjustment. Comparable patterns have been reported in smaller clinically defined cohorts, where higher cesarean section rates were largely observed in conjunction with placental complications or disease severity [12, 13].

These observations are consistent with population-based studies demonstrating attenuation after accounting for maternal age, infertility, mode of conception and placental pathology [8, 28]. In the present cohort, all cases of placenta previa were delivered by cesarean section, underscoring that cesarean delivery represents a downstream consequence of abnormal placentation [5].

## Conclusion

In this analysis, endometriosis was associated with an increased risk of placenta previa, whereas no independent associations were observed with preterm birth or cesarean delivery after adjustment. These findings suggest that endometriosis may be linked to abnormal placentation rather than to a generalized obstetric risk profile. Together, the results underscore the importance of careful cohort definition and conception-mode stratification, when evaluating pregnancy outcomes associated with endometriosis.

## Supplementary Information

Below is the link to the electronic supplementary material.

Supplementary file1 (DOCX 24 KB)

## Data Availability

The datasets generated and analyzed during the current study are not publicly available due to patient confidentiality, but are available from the corresponding author on reasonable request.

## Abbreviations

BMI: Body mass index
CI: Confidence interval
IQR: Interquartile range
MAR: Medically assisted reproduction
OR: Odds ratio
aOR: Adjusted odds ratio
