# Supplementary material for: Endometriosis and adverse pregnancy outcomes in primiparous women: a retrospective cohort study
Source: Arch Gynecol Obstet. 2026 May 20;313(1):196. doi: 10.1007/s00404-026-08465-5 (PMC13190462; doi:10.1007/s00404-026-08465-5)
Supplement: Supplementary file 1 — Supplementary file1 (DOCX 24 KB) [file 404_2026_8465_MOESM1_ESM.docx]

**Table S1. Clinical characteristics of primiparous women with endometriosis (n = 118)**

| **Characteristic** | **Value** |
| --- | --- |
| Age at endometriosis diagnosis, years, median (IQR) | 29.5 (25–33) |
| Endometriosis diagnosed before pregnancy, n (%) | 113 (95.8) |
| Endometriosis diagnosed at delivery, n (%) | 5 (4.2) |
| Interval between endometriosis diagnosis and first delivery, months, median (IQR) | 25.4 (16.0–44.6) |
| Prior pregnancy loss/termination, n (%) | 31 (26.3) |
| Endometriosis phenotype, n (%) |  |
| – Peritoneal endometriosis | 65 (55.1) |
| – Ovarian endometrioma | 56 (47.5) |
| – Deep infiltrating endometriosis | 53 (44.9) |
| Adenomyosis, n (%) | 29 (24.6) |
| rASRM classification documented, n (%) | 35 (29.7) |
| – Stage I | 6 (17.1) |
| – Stage II | 13 (37.1) |
| – Stage III | 5 (14.3) |
| – Stage IV | 11 (31.4) |
| Complete #Enzian classification available, n (%) | 55 (46.6) |

Data are presented as median (IQR) or n (%). Endometriosis was laparoscopically and histologically confirmed prior to the index pregnancy in all but five women. Endometriosis phenotypes were not mutually exclusive. IQR, interquartile range.

**Table S2. Additional pregnancy outcomes in primiparous women with and without endometriosis**

| **Outcome** | **Endometriosis (n = 118)** | **Controls (n = 15,915)** | **P-value** |
| --- | --- | --- | --- |
| Birthweight, g, mean (SD) | 3171 (573) | 3143 (691) | 0.912 |
| Sex of the newborn, n (%) |  |  | 0.279 |
| – Female | 50 (42.4) | 7646 (48.0) |  |
| – Male | 66 (55.9) | 8237 (51.8) |  |
| Gestational diabetes, n (%) | 8 (6.8) | 1138 (7.2) | 0.876 |

Data are presented as mean (SD) or n (%). P-values were calculated using chi-square test for categorical variables and Mann–Whitney U test for continuous variables. SD, standard deviation.

**Table S3. Sensitivity analysis excluding women with adenomyosis**

| **Group** | **Placenta previa, n (%)** |  |
| --- | --- | --- |
| Endometriosis (n = 89) | 3 (3.4) |  |
| Controls (n = 15,915) | 83 (0.5) |  |
|  |  |  |
| **Adjusted analysis** |  |  |
| **Outcome** | **Adjusted OR (95% CI)** | **P-value** |
| Placenta previa | 4.08 (1.21 – 13.75) | 0.023 |

Adjusted for maternal age at delivery and medically assisted reproduction.

**Table S4. Distribution of gestational age subgroups among preterm births**

| Gestational age (weeks) | Endometriosis  (n = 16) | % | Controls  (n = 2,279) | % |
| --- | --- | --- | --- | --- |
| < 28+0 | 0 | 0.0 | 313 | 13.7 |
| 28+0 – 31+6 | 1 | 6.3 | 299 | 13.1 |
| 32+0 – 33+6 | 5 | 31.3 | 290 | 12.7 |
| 34+0 – 36+6 | 10 | 62.5 | 1,377 | 60.4 |

One control with missing gestational age data was excluded from the subgroup distribution; therefore the control denominator in this table is n = 2,279 instead of n = 2,280.
